# Structure–function relationships of the antigenicity of mycolic acids in tuberculosis patients

**DOI:** 10.1016/j.chemphyslip.2010.09.006

**Published:** 2010-11

**Authors:** Mervyn Beukes, Yolandy Lemmer, Madrey Deysel, Juma’a R. Al Dulayymi, Mark S. Baird, Gani Koza, Maximiliano M. Iglesias, Richard R. Rowles, Cornelia Theunissen, Johan Grooten, Gianna Toschi, Vanessa V. Roberts, Lynne Pilcher, Sandra Van Wyngaardt, Nsovo Mathebula, Mohammed Balogun, Anton C. Stoltz, Jan A. Verschoor

**Affiliations:** a Department of Biochemistry, University of Pretoria, South Africa; b Department of Chemistry, University of Pretoria, South Africa; c Department of Infectious Diseases, University of Pretoria, South Africa; d School of Chemistry, University of Wales, Bangor, United Kingdom; e Department of Molecular Biomedical Research, Molecular Immunology Unit, Gent University, Belgium

**Keywords:** Mycolic acids, Cholesterol, Monoclonal antibodies, Tuberculosis, Diagnostics, Antigenicity

## Abstract

Cell wall mycolic acids (MA) from *Mycobacterium tuberculosis* (*M.tb*) are CD1b presented antigens that can be used to detect antibodies as surrogate markers of active TB, even in HIV coinfected patients. The use of the complex mixtures of natural MA is complicated by an apparent antibody cross-reactivity with cholesterol. Here firstly we report three recombinant monoclonal scFv antibody fragments in the chicken germ-line antibody repertoire, which demonstrate the possibilities for cross-reactivity: the first recognized both cholesterol and mycolic acids, the second mycolic acids but not cholesterol, and the third cholesterol but not mycolic acids. Secondly, MA structure is experimentally interrogated to try to understand the cross-reactivity. Unique synthetic mycolic acids representative of the three main functional classes show varying antigenicity against human TB patient sera, depending on the functional groups present and on their stereochemistry. Oxygenated (methoxy- and keto-) mycolic acid was found to be more antigenic than alpha-mycolic acids. Synthetic methoxy-mycolic acids were the most antigenic, one containing a *trans*-cyclopropane apparently being somewhat more antigenic than the natural mixture. *Trans*-cyclopropane-containing keto- and hydroxy-mycolic acids were also found to be the most antigenic among each of these classes. However, none of the individual synthetic mycolic acids significantly and reproducibly distinguished the pooled serum of TB positive patients from that of TB negative patients better than the natural mixture of MA. This argues against the potential to improve the specificity of serodiagnosis of TB with a defined single synthetic mycolic acid antigen from this set, although sensitivity may be facilitated by using a synthetic methoxy-mycolic acid.

## 1 Introduction

South Africa currently has the highest per capita incidence of TB in the world. In 2007 alone 112,000 people died of TB in South Africa, of whom 94,000 were co-infected with HIV (WHO, 2009). One of the biggest challenges facing clinicians is the time it takes to accurately diagnose TB. Currently, using the conventional methods, it takes on average 4 weeks to diagnose TB, which leads to a delay in treatment of the disease. Two thirds of TB deaths could be prevented by early diagnosis. With fast diagnosis patients could be put on anti-TB therapy immediately and become non-infective within a few days. With the current methods of diagnosis, patients with persistent symptoms have to remain in quarantine for several weeks while awaiting the results. During this time, they can infect the medical staff, their next of kin or anyone with whom they share a closed area, such as in public transport. With MDR and XDR TB on the increase, this threatens to spread an almost incurable disease among hospital staff and the communities that can be fatal within 2 months. The need for a fast, reliable diagnostic tool for TB is therefore high, especially in high HIV incidence populations (Wood, 2007).

Immunodiagnostic assays detecting pathogen related antibodies in patient sera with active TB disease are an attractive alternative for rapid diagnosis. An array of mycobacterial cell wall components have been considered as antigens for surrogate marker antibodies for TB (Fujiwara et al., 1999; Lyashcenko et al., 1998; Nabeshima et al., 2005). Antigenic activity of mycolic acids (MA) and their glycolipid derivatives such as the lipid extractable trehalose mono- or dimycolates, TMM or TDM respectively (cord factors) has been reviewed recently (Sekanka et al., 2007). Of all the antigens prevalent in the cell wall of the mycobacteria that may be considered for use in TB serodiagnosis, MAs provide a special opportunity due to their abundance, variability among different species of *Mycobacterium* and the unique way that they communicate their presence to the immune response of the host (Sekanka et al., 2007; Shui et al., 2007; Yuan et al., 1997). The ability of MAs to elicit CD4-, CD8-double negative T cells by means of their presentation on CD1b proteins of dendritic cells (Beckman et al., 1994) may well be the reason that antibody binding to MAs in AIDS patients with even very low CD4 T cell counts is maintained, relative to other patients that are not infected with HIV, or have normal CD4 T cell counts (Schleicher et al., 2002). Pan et al. have shown that the most determining antigenic part of the cord factor antigen is the MA (Pan et al., 1999).

The use of MA antigens to detect antibodies as surrogate markers for TB diagnosis was shown to be feasible in ELISA assays (Pan et al., 1999; Schleicher et al., 2002), albeit of limited accuracy. One complication was the apparent cross-reactivity of TB patient serum antibodies between MAs and cholesterol, most likely due to a shared structural feature between cholesterol and a folded form of MA, as both could be liganded by Amphotericin B, a cholesterol binding drug (Benadie et al., 2008). A biosensor approach, the MARTI-test (*M*ycolic acids *A*ntibody *R*eal-*T*ime *I*nhibition), using free natural mixtures of MAs in liposomes as antigens in a competitive binding assay showed improved accuracy (Lemmer et al., 2009; Thanyani et al., 2008). This test can diagnose TB within four hours of sampling by detecting anti-MA antibodies as immune surrogate markers for active disease even in HIV infected patients. Although the use of the inhibition of binding of antibodies in a real-time immunoassay seemed to practically solve the problem of cross-reactivity between MAs and cholesterol, it is expected that better resolution between TB positive and TB negative patient sera will be achieved if the nature of the cross-reactivity is better understood. A structure-activity investigation of the antigenicity of MAs and cholesterol may identify an individual synthetic MA which is more selectively antigenic than the natural mixtures.

MAs comprise a large number of various structures within and among *Mycobacterium* species and in a few other genera. In *M. tuberculosis*, they consist mainly of alpha-, keto- and methoxy-MA subclasses, each containing mixtures of homologues of varying chain length and, in some cases different stereochemistry around the functional groups in the main (mero-) chain (Dobson et al., 1985). They are present either bound to the cell wall as penta-arabinose tetramycolates or as sugar esters (e.g., trehalose dimycolates and trehalose monomycolates). There is increasing evidence of the importance of some natural free MAs (Ojha et al., 2008). Whether all, a few, or one of these MAs is detected as antigens by TB patient antibodies is not clear and is one focus of this report. Pan et al. (1999) indicated that the methyl esters of homologous mixtures of natural methoxy-MAs are more antigenic than those of the keto-MA or the non-oxygenated alpha-MA. A more sensitive and specific diagnostic assay could possibly be developed by making use of specific stereoisomers of single chain lengths of synthetic MA subclasses instead of using natural mixtures of MA. Because different MA subclasses dominate in certain stages of the growth of mycobacteria or stage of disease, it could also be that a specific synthetic MA antigen could provide more reliable data, reveal information on the progress of the disease and be better able to distinguish between TB positive and TB negative patient sera.

**Fig. 1:**
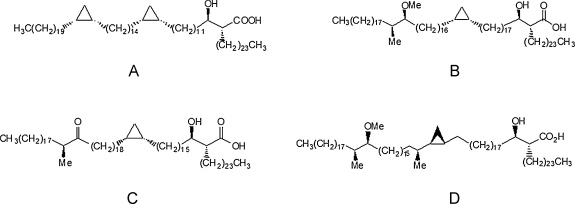
Structures of the prominent homologues of three main classes of MA of *Mycobacterium tuberculosis*. **A**, alpha-; **B**, methoxy-; **C**, keto-mycolic acid; and **D**, the α-methyl-*trans*-cyclopropane form of natural methoxy-mycolic acid.

The three major classes of MA are exemplified by structures **A**–**C** (Fig. 1). In each of these, the stereochemistry of the hydroxy acid part is *R*,*R*- and that of the methoxy-methyl fragment in B is reported to be *S*,*S*. The α-methylketone of **C** is also apparently of *S*-stereochemistry, although often in the isolation of such compounds from cells by chemical hydrolysis this centre is epimerized to a mixture of *R*- and *S*-forms. The absolute stereochemistry of the *cis*-cyclopropane remains uncertain, although if a common intermediate is involved in producing methoxy-, hydroxy-, keto- and cyclopropane functionalities, it will be as shown in **A**–**C** (see e.g., Koza et al., 2009a). More recently it has become clear that hydroxy-MAs are probably intermediate in the formation of methoxy- and keto-MAs and indeed some examples have been detected directly (Quémard et al., 1997; Dubnau et al., 1997). In a number of cases, the proximal *cis*-cyclopropane is replaced by an α-methyl-*trans*-cyclopropane as in, for example, **D**. Generally such compounds will be present together with the corresponding *cis*-cyclopropane in the complex mixture of different classes and different homologues (chain lengths) of MA extracted from cells. Even when the MA extracted from cells is separated into α-, methoxy- and keto-classes, the *cis*- and *trans*-cyclopropanes are generally not separated.

The chemical syntheses of MAs representative of various subclasses that appear in the cell wall of *M.tb* and containing both *cis*-cyclopropanes and α-methyl-*trans*-cyclopropanes have only been reported since 2005 (Al Dulayymi et al., 2005, 2006a,b, 2007; Koza and Baird, 2007; Koza et al., 2009b). Indeed, although there are a number of reports on the biological effects of types of MA isolated from cells, we are unaware of any experiments which distinguish the role of *cis*- and *trans*-cyclopropanes directly.

The objectives of this study were: (i) to better understand the cross-reactivity of MA antigens with cholesterol by selecting monoclonal antibody specificities from a recombinant chicken immunoglobulin gene library that might or might not cross-react between these two antigens and (ii) to determine the structural features of MA required for antigenicity using TB positive and TB negative pooled human serum samples in ELISA. This knowledge may be useful to improve TB serodiagnostic tests that are based on the detection of antibodies to MAs as surrogate markers of active TB.

## 2 Materials and methods

### 2.1 Preparation of methyl ester of natural mycolic acid mixture

Mycolic acid from the *M. tuberculosis* virulent strain was purchased from Sigma–Aldrich, batch M4537. The acid was converted into the corresponding methyl ester. MA (100 mg to 0.1 mmol) was dissolved in a mixture of toluene: methanol (5:1, 18 ml). Thereafter a 2 M solution in n-hexane of trimethylsilyldiazomethane (0.2 ml, 0.4 mmol, 4 mol eq.) was added. This addition was repeated another 3 times, every 45 min (0.1 ml, 0.2 mmol, 2 mol eq.). The reaction was monitored by TLC using 4:1 hexane:ethyl acetate solution. After stirring for 72 h, the reaction was quenched by evaporation of the volatiles to give a white residue. This was dissolved in dichloromethane (15 ml) and water (10 ml) was added. The water layer was washed with dichloromethane (2× 10 ml). The combined organic layers were dried and the solvent evaporated to give the desired MA methyl ester (me-MA). The HNMR and CNMR spectra of this ester were consistent with those reported (Laval et al., 2001).

### 2.2 Fluorescent labelling of natural mycolic acids

MAs (Sigma–Aldrich) were esterified to 5-bromomethyl-fluorescein (5BMF) as described by Lemmer et al. (2009).

### 2.3 Preparation of synthetic mycolic acids

Mycolic acids representative of the major homologues present in *M.tb* were prepared as previously described (Al Dulayymi et al., 2005, 2006a,b, 2007; Koza and Baird, 2007; Koza et al., 2009b) or by simple variations of those methods. Full details of all the known compounds have been reported already; corresponding details for the unpublished isomers are provided as supplementary information in Table 1.

### 2.4 Generation of recombinant monoclonal scFv

#### 2.4.1 Phage display antibody library

A naive semi-synthetic chicken phage display library was used (Van Wyngaardt et al., 2004). The library contains recombinant filamentous bacteriophages displaying scFv antibody fragments. These fragments were derived from combinatorial pairings of chicken V_H_ and V_L_ immunoglobulin domains. V_H_ and V_L_ domains are linked by an interpeptide segment consisting of the sequence (GGGGS)_3_, enabling a fold typical of single variable fragments.

#### 2.4.2 Phage display antibody selection

A selection of the phages displaying MA reactive scFv's was conducted by several panning rounds. Maxisorp immunotubes (Nunc-Immuno Tubes, Nunc, Denmark) were coated with 100 μg/ml mycolic acid (Sigma–Aldrich) dissolved in distilled hexane, after which the hexane was allowed to evaporate at room temperature. Coated immunotubes were briefly washed with phosphate buffered saline (PBS, pH 7.4), then blocked with 2% skimmed milk in phosphate buffered saline (2% MPBS) for 60 min. Tubes were then exposed to 10^12^ transforming units of the phage library in 2% MPBS, 0.1% Tween-20 buffer for 2 h. Unbound phage was removed by 10× washing with PBS containing 0.1% Tween-20 followed by a further 10× wash with PBS to remove the Tween-20. Bound phage was eluted with 100 mM triethylamine and neutralized with 1 M Tris, pH 7.4. For enrichment *Escherichia coli* TG1 was infected with eluted phages, grown at 30 °C in 2× TYG broth (TY broth supplemented with 2% glucose) containing 100 μg/ml ampicillin, and rescued with M13-K07 helper phage (Invitrogen). Panning was repeated four times.

#### 2.4.3 Screening of mycolic acid specific phage clones

Following the final panning, individual ampicillin resistant *E. coli* TG1 colonies were selected for further characterization. Colonies were grown in 2× TYG broth supplemented with 100 μg/ml ampicillin in 96-well Microtitre plates at 30 °C. Phages were rescued as described previously (Van Wyngaardt et al., 2004). Phage clones were screened by enzyme-linked immunosorbent assay (ELISA) carried out with MA coated (50 μg/ml) microtitre plates (Maxisorp, Nunc, Denmark). Coating was done by adding 50 μl of 100 μg/ml MA in hexane into each well and evaporating it overnight at room temperature. Wells were briefly washed with PBS, and blocked with 300 μl of 2% MPBS for 60 min. Phage containing supernatants (25 μl) were mixed with blocking solution (25 μl), added to each well, and incubated for 60 min at 30 °C. Wells were washed three times with PBS-0.1% Tween-20. Mouse monoclonal antibody B62-FE2, specific for M13 filamentous phage, in 2% MPBS-0.1% Tween-20 (50 μl) was added to each well and further incubated for 60 min at 30 °C. Bound phages were detected using rabbit anti-mouse IgG antibody conjugated with horseradish peroxidase (HRP). Signals were developed with 3,3′,5,5′-tetramethylbenzidine using the 1-stepUltra TMB ELISA substrate solution according to manufacturer's instructions. Plates were read using a Multiskan Ascent (Thermo Labsystems) plate reader at a wavelength of 450 nm.

#### 2.4.4 Production and purification of mycolic acid reactive scFv

Selected anti-MA phage obtained from *E. coli* TG1 clones was used to infect *E. coli* HB2151 to obtain soluble scFv. Single colonies were grown to an OD_600_ of 0.9 in 2× TYG broth supplemented with 100 μg/ml ampicillin at 37 °C. ScFv expression was induced with isopropyl β-d-thiogalactosidase (IPTG; 1 mM) and the culture further incubated at 30 °C overnight, in glucose free media. Soluble scFv was extracted with 1× TES buffer from the periplasm as previously described (Hugo et al., 2002). ScFv was further affinity purified using an anti *c-myc* tag mAb commercially denoted as 9E10 IgG. The column was prepared by immobilising 9E10 IgG onto aminoLink Plus gel (Pierce) according to manufacturer's instructions. Periplasmic extracts were applied and after washing with PBS, bound scFv was eluted with 100 mM triethylamine and neutralised with 1 M Tris, pH 7.4. Eluted scFv was dialyzed against 1× PBS, pH 7.4 at a MW cut-off of 10 kDa. Samples were concentrated using a Macrosep^®^ ultrafiltration device (Pall life sciences, USA) and protein concentrations determined with a BCA protein detection kit (Pierce, USA), according to the manufacturer's instructions. Purified scFv was stored at −20 °C until further use.

### 2.5 Enzyme-linked immunosorbent assay (ELISA)

#### 2.5.1 Analysis of the methyl ester and free acid of mycolic acids

For coatings done in PBS, methyl MA (me-MA) or free MA (250 μg) was dissolved in 1× PBS (4 ml, pH 7.4) and placed on the heat block at 90 °C for 20 min. One vial of 1× PBS (4 ml) served as control. The solutions were vortexed for 30 s before sonifying for 2 min using a Virsonic sonifier at output of 2. The warm solutions were subsequently loaded onto the ELISA plates (50 μl per well) and the presence of oily drops viewed under a light microscope. The plates were kept at 4 °C overnight in plastic bags. For the coatings done using hexane as coating solution, the lipid samples (250 μg) were dissolved in hexane (4 ml, distilled) and vortexed for 30 s. One vial of hexane (4 ml) served as control. Solutions were coated using a Hamilton syringe (50 μl/well) and the liquid was loaded in the centre of the wells. Lipid was visible as a circular waxy layer after 2 h of evaporation of the hexane at room temperature. The plates were then stored in plastic bags at 4 °C overnight. The human sera used for analysis of the methyl ester and free MA samples were a pooled TB positive patient serum and a pooled TB negative patient serum at a 1:20 dilution of serum. The pooled TB pos sample was created by pooling the sera of six patients, three TB positive/HIV positive (TB pos/HIV pos) and three TB positive/HIV negative (TB pos/HIV neg) randomly selected from a collection used for another study (Schleicher et al., 2002) in which it was shown that HIV or its state of progression to AIDS did not affect binding activity of antibodies to natural mycolic acids. TB neg patients were hospitalized for medical conditions other than tuberculosis, but showed no evidence of active tuberculosis. For ELISA the PBS lipid coated plates were aspirated and then blocked with 0.5% Casein/PBS (400 μl/well), while the dry hexane coated plates were directly blocked with 0.5% Casein/PBS (400 μl/well). After 2 h, the blocking buffer was aspirated and serum (1:20 dilution in 0.5% Casein/PBS, pH 7.4) was added to the plate (50 μl/well). After 1 h of serum incubation, the wells were washed three times with a Well Wash4 ELISA washer (Labsystems) and flicked out before adding the goat anti-human Immunoglobulin G (IgG) peroxidase conjugate (whole molecule) for 30 min at room temperature. Subsequently, plates were washed three times and flicked out before adding the OPD substrate solution (50 μl/well). Absorbancies were measured with a SLT 340 ATC photometer at 450 nm with a reference filter at 690 nm at 30 min and 50 min for hexane and PBS coated plates respectively. Background binding of serum to PBS or hexane was corrected for by subtracting each serum response to PBS or hexane from the antibody binding values obtained to the coated lipid antigens. Statistical comparisons of ELISA results were performed using the student *t*-test at a confidence level of 95%.

#### 2.5.2 Analysis of the synthetic mycolic acids

To coat the ELISA plates with the different synthetic mycolic acids subclasses and the natural mycolic acids to which they were compared, the lipids were dissolved in hexane (3 μg/50 μl) and vortexed 1 min, heated (at ∼85 °C) for a minute and allowed to stand at room temperature for 15 min. Hexane coating as such served as a control. The ELISA plates were coated with the different mycolic acids at 50 μl per well by application to the well using Hamilton syringes. The lipids were visible as a waxy coating after 2 h of evaporation at RT. Plates were stored in a plastic bag at 4 °C overnight. ELISA was done as described in Section 2.5.1 Background binding of the serum to the plate was corrected for by subtracting the average binding signal of antibody to MA from that registered for the hexane coated wells. The results obtained were analysed by the making use of the Student's *t*-test for statistical significance.

#### 2.5.3 Characterization of scFv's binding specificity of mycolic acids by sandwich ELISA

Purified scFv's were tested for their binding activity using a sandwich ELISA. Maxisorp immunoplates were coated with MA as described above. Plates were blocked with 2% MPBS for 60 min at 30 °C followed by a brief washing step with PBS. ScFv samples (25 μl) were mixed with 2% MPBS (25 μl), added to the wells and incubated for 60 min. Unbound scFv was removed by 3× washing with PBS-0.1% Tween-20. Anti c-myc monoclonal antibody (AbD serotec, UK) conjugated with HPR was used to detect bound scFv fragments. Signals were developed with 3,3′,5,5′-tetramethylbenzidine using the 1-stepUltra TMB ELISA substrate solution according to manufacturer's instructions. Plates were read using a Multiskan Ascent (Thermo Labsystems) plate reader at a wavelength of 450 nm.

## 3 Results and discussion

### 3.1 Antibody recognition of natural mycolic acids and ester derivatives thereof

The strongly hydrophobic nature of MA makes them insoluble in water and water miscible organic solvents. Their recognition by water soluble antibodies in diluted serum is therefore somewhat enigmatic and requires proof that antibody binding is not due to non-specific hydrophobic antibody adsorption to the MA coated surface.

**Fig. 2:**
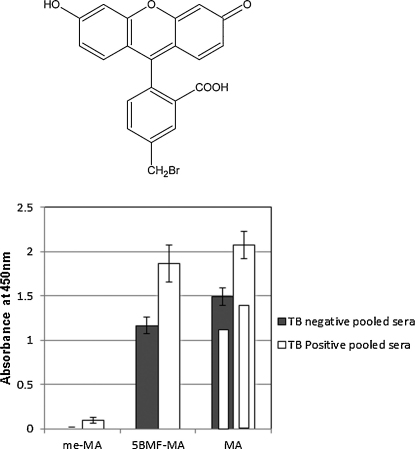
Human patient antibody recognition of natural Mtb MAs (MA mix), methyl ester of natural MA (me-MA) and natural MA fluorescein ester (5BMF-MA, prepared from natural MA mix and 5BMF (structure)) measured with ELISA. Pooled TB positive and pooled TB negative sera were tested on the MA antigen derivatives coated from hexane. Inner bars within the MA mix bars indicate the signals when coating was done from hot PBS instead of hexane. The error bars indicate the standard deviation. The 2.5 d rule was applied to remove outliers. *n* = min 14, max 16.

In Fig. 2, the specificity of interaction of TB positive and negative sera with coated MA antigens is demonstrated. Hexane appears to be the better antigen coating solution compared to hot PBS. The MA methyl-ester (me-MA) is not recognized by antibodies, whereas the free MA (MA mix) is recognized by both TB pos and TB neg patient sera, but more strongly with TB pos sera. This supports the hypothesis that the ELISA antibody binding signal is due to recognition of an antigen consisting of one or more MAs, in which the hydroxyl group of the free MA-carboxylic acid probably participates in inter- or intramolecular stabilization of a specific antigen conformation. When the MA is fluoresceinated by esterification of its carboxylic acid, the antibody binding signal is not significantly affected, possibly due to the fact that fluorescein substitutes a free carboxylic acid group in close proximity to where the fluorophore compromises the free carboxylic acid of mycolic acid by ligandation (Fig. 2). These results corroborate those of Lemmer et al. (2009), who reported similar results when MA and fluoresceinated MA were presented on immobilized liposomes to patient sera in a surface plasmon resonance biosensor.

### 3.2 Monoclonal scFv antibody fragment recognition of mycolic acid and cholesterol

**Fig. 3:**
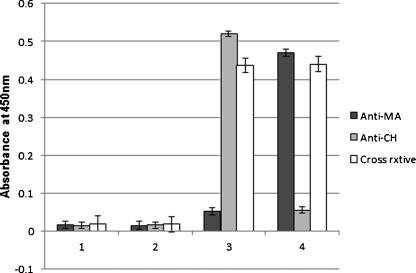
Chicken scFv antibody fragment recognition of MA (4 = MA mix) and cholesterol (3 = CH) with ELISA. Three scFv specifities were identified, which were denoted Anti-MA (black bars), Anti-CH (grey bars) and Cross rxtive (blank bars). MA and cholesterol antigens were coated from hexane, while results on hexane (1 = Hexane) and PBS (2 = PBS) sham coated wells are indicated as well. The error bars indicate the standard deviation of four repeats.

The high antibody binding signal with human TB negative sera against MA, here again shown in Fig. 2, was previously speculated to be due at least in part to cross-reactivity of the antibodies with cholesterol (Schleicher et al., 2002), an idea that was later supported by showing that both MA and cholesterol were recognized equally well by Amphotericin B in an evanescent field biosensor (Benadie et al., 2008). The cross-reactivity could be due to a mixture of monospecific anti-cholesterol and anti-MA antibodies in the sera, or due to a true cross-reactivity where a particular antibody specificity could recognize both MA and cholesterol. It is known that all humans have anti-cholesterol antibodies in their blood circulation (Swartz et al., 1988), which may at least in part explain the high antibody activity to MAs in TB negative patients. To test what mechanisms are possible for the cross-reactivity, scFv fragments expressed from a chicken antibody gene library were screened for specific binders to cholesterol and MA. Chickens, similar to humans, express a specialized MA-presenting CD1 (chCD1-1) on their antigen presenting cell populations (Dvir et al., 2010). Three different specificities were detected and worked up from the phage display system into monovalent, monoclonal scFv fragments. The mono-specific anti-cholesterol scFv was dubbed anti-CH, while two scFv specificities were generated against MA: one monospecific (Anti-MA) and one cross-reactive with cholesterol (Cross rxtive). Fig. 3 shows the characterization of these three scFv's with ELISA. The fact that a single monoclonal, monovalent cross-reactive scFv could be found with binding affinity against both cholesterol and MA corroborates the conclusion reported by Benadie et al. (2008) that cholesterol and MA share some antigenic structural properties and may explain why TB negative sera recognize cholesterol as well as they do MA. On the other hand, the finding that an scFv against MAs could be found that does not cross-react with cholesterol (Anti-MA) and vice versa (Anti-CH), means that anti-MA antibodies may be induced during tuberculosis that are not merely anti-cholesterol antibodies with increased binding activity. This provides a broader perspective on why the results in Fig. 2 show higher antibody binding activity with TB pos patient sera than with TB neg sera as was shown before with larger numbers of human patient sera from the same collection (Schleicher et al., 2002; Thanyani et al., 2008).

### 3.3 Antibody recognition of synthetic mycolic acids

MAs in *M.tb* contain an *R,R*-α-alkyl-β-hydroxy acid. The main branch, known as the meromycolate moiety, contains two functionalities at the so called distal and proximal positions (Sekanka et al., 2007). The proximal position is usually either a *cis*- or an alpha-methyl-*trans*-cyclopropane, while the distal functionality is usually a *cis*-cyclopropane or one of several oxygenated functional groups including β-methyl-hydroxyl-, β-methyl-methoxy- and β-methyl-keto-groups. MA derivatives were chemically synthesised that were representative of four MA subclasses, namely methoxy-MA, hydroxy-MA, keto-MA and alpha-MA (Table 1). The response of pooled TB positive and TB negative patient sera towards the different synthetic MAs was compared to that obtained towards natural free MA and isolated natural alpha MA in ELISA. The importance of the stereochemistry of the merochains of MAs for antigenic activity was studied by using different single stereoisomers of chemically synthesised MAs as antigens. Hexane was used as solvent to coat the plates with the MAs.

**Fig. 4:**
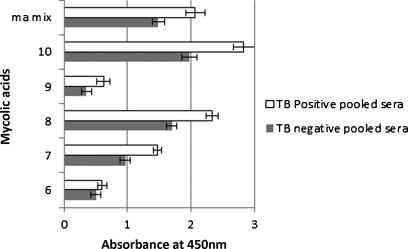
Comparable ELISA signals of pooled TB positive and TB negative sera to synthetic methoxy-mycolic acids. The error bars indicate the standard deviation. The 2.5 d rule was applied to remove outliers. *n* = min 14, max 16.

**Fig. 5:**
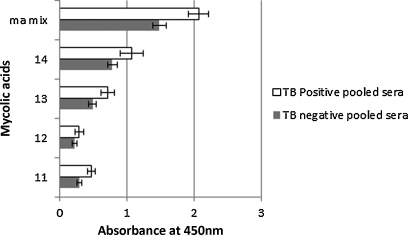
ELISA antibody binding signals of pooled TB positive and TB negative sera to synthetic hydroxymycolic acids. The error bars indicate the standard deviation. The 2.5 d rule was applied to remove outliers. *n* = min 15, max 16.

**Fig. 6:**
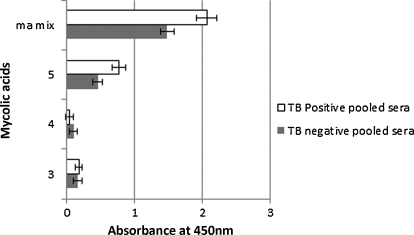
ELISA antibody binding of pooled TB positive and TB negative sera to synthetic keto-mycolic acids. The error bars indicate the standard deviation. The 2.5 d rule was applied to remove outliers. *n* = min 15, max 16.

**Fig. 7:**
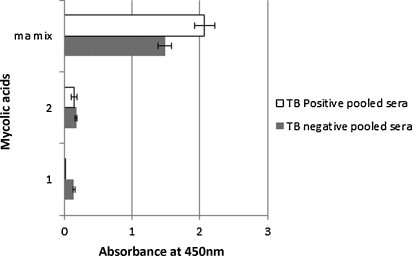
ELISA antibody binding signals of TB positive and TB negative sera to synthetic alpha mycolic acids. MA-mix = the natural mixture of mycolic acids, 13, 14 = chemically synthetic alpha MA with structures indicated. The error bars indicate the standard deviation. The 2.5 d rule was applied to remove outliers. *n* = min 8, max 16.

In general, synthetic methoxy-MA bound most strongly (Fig. 4), followed in descending order by hydroxy-MA (Fig. 5), keto-MA (Fig. 6) and alpha-MA (Fig. 7). The exact stereochemistry of each sub-type, i.e. the precise spatial arrangement of the functional groups, also appeared to be important. The observation that the oxygenated mycolic acids are more antigenic than the alpha-MA confirms a previous report by Pan et al. (1999), although they used the methyl esters of the MA subclasses in ELISA, a result which contrasts our finding that the methyl esters are not antigenic (Fig. 2). The packing of mycolic acids in a Langmuir monolayer has previously been shown to differ between alpha-, keto- and methoxy-MA subclasses. Keto-MA tended more towards a W-shaped configuration with exceptional rigidity in monolayers, whereas methoxy- and alpha-MA exhibited a more flexible conformation towards variation of experimental parameters (Villeneuve et al., 2005, 2007). Thus, the packing of MA is influenced by the orientation of the functional groups that induce different conformations for interaction with antibodies in sera. We propose that the antibody binding to mycolic acids with TB negative sera is most likely due to the presence of anti-cholesterol antibodies, known to exist in all humans (Bıro et al., 2007; Swartz et al., 1988) that cross-react with MA.

Antibody binding to the natural MA mixture, as well as several of the synthetic MAs was observed with both TB positive and TB negative patient sera, while some synthetic MAs appeared to have little or no antigenic activity. Although TB positive sera generally gave better binding to the antigenic MAs than TB negative sera, no single antigenic MA was significantly better able to differentiate between TB positive and TB negative sera than the natural mixture of MAs could. This could in principle mean that the synthetic MAs tested were antigenic primarily to the antibodies that respond to both MA and cholesterol. The fact that TB positive patient sera statistically score higher than TB negative patient sera in recognition of MA in ELISA could then explained simply by a higher concentration or affinity of the anti-MA/cholesterol antibodies in TB patient sera, even though monospecific antibody activity to mycolic acids has been found to exist at least in the germ-line antibody gene repertoire of chickens (Fig. 3).

The synthetic methoxy-MA subclass had the highest binding to the antibodies of the four synthetic subclasses tested, followed by hydroxy-, keto- and alpha-MA (Figs. 4–7). The stereochemistry of the methoxy group and the cyclopropane is important for the recognition by antibodies in the sera. Even small changes in the stereochemical arrangement of the groups influenced the amount of binding observed. As seen from Fig. 4, the antibody binding signal of *R,R*-methoxy-methyl-*cis*-cyclopropane MA configuration (No. 8) most closely resembles the response towards the natural mixture of MA. A change of the configuration of either the *cis*-cyclopropane (No. 7), or the methoxy-methyl-fragment to *S,S* (No. 9) reduced the binding signal by approximately half. If the more weakly antigenic *S,R*-*cis* configuration of the cyclopropane is combined with the *S,S*-methoxy configuration (No. 6), the signal is once again halved, even though the *S,S*-methoxy configuration is the reported stereochemistry in natural compounds (Al Dulayymi et al., 2007). However, when the cyclopropane group assumed the methyl-*trans*-configuration as in structure No. 10, in this case in association with the reported natural *S,S* configuration around the methoxy-methyl fragment, an antigenicity signal was obtained that was even higher than that obtained for the natural MA mixture (*P* < 0.01). This shows beyond doubt that the stereochemical configuration of the two functional groups on the mero-chain of the methoxy-MA influences the way in which they are recognised by antibodies in human serum. It remains to be determined whether other combinations of absolute configuration of the methoxy-methyl fragment and the methyl-*trans*-cyclopropane will provide an even more antigenic MA.

The synthetic hydroxy-MAs (Fig. 5), which are the likely precursors of both methoxy- and keto-MA (Yuan and Barry, 1996; Yuan et al., 1997; Asselineau et al., 2002) all attracted weaker antibody binding compared to the natural MA mixture. The methyl-*trans* configuration of the proximal cyclopropane group appears to be a pre-requisite for antigenicity of the hydroxy-MAs (Nos.13 and 14, compared to Nos. 11 and 12, Fig. 5). The hydroxy-methyl fragment in the *R,R* conformation (No. 14) is more antigenic than the supposed natural *S,S* configuration (No. 13) with statistical significance, *P* < 0.01, as was found with the methoxy group stereochemistry (Fig. 4).

Like the hydroxy-MAs, keto-MAs also require a proximal cyclopropane in the methyl-*trans*-cyclopropane configuration (No. 5, Fig. 6) to be antigenic, compared to the two *cis*-cyclopropane configurations (Nos. 4 and 3, Fig. 6) that did not show any significant antigenic activity. In these cases, a mixture of epimers at the chiral centre adjacent to the keto-group was tested; it is possible that *R* and *S*-isomers show different antigenicity. Thus in all the oxygenated MAs (i.e., methoxy-, hydroxy- and keto-MA), the methyl-*trans*-cyclopropane configuration provides for the best antigenic functionality.

Two synthetic alpha MAs (Fig. 7, Nos.1 and 2) were compared to the natural MA mix to determine their relative antigenicity. The antibody binding signal to the synthetic alpha MA failed to distinguish between TB pos and TB neg patient sera compared to the natural MA mix. The synthetic MA gave such low antibody binding signals, that nothing could be learned from the variations in the stereochemistries of the distal and proximal cyclopropanes on the antigenicity of alpha-MA. It must be noted that the stereochemistry of *cis*-cyclopropanes in alpha-mycolates remains to be proven. However, if a common synthetic intermediate is involved in the production of the different functionalities and this is the same for both proximal and distal substituents, the natural stereochemistry will be as in **1**.

## 4 Conclusion

MAs have been shown to play an important role in the virulence of tuberculous mycobacteria. Not only do they act as pathogen associated molecular patterns (PAMP) for induction of murine innate immunity (Korf et al., 2005), but they are also able to reprogramme murine macrophages to modulate their inflammatory activity (Korf et al., 2006). That these responses towards free MA administration may be related to the functional groups expressed on the merochain is inferred by findings such as that mutants of *M. tuberculosis* that have no oxygenated mycolic acids are of reduced virulence in mice (Dubnau et al., 2000), that mutants without the ability to *trans*-cyclopropanate their oxygenated MAs are hypervirulent (Rao et al., 2006), and that the trehalose dimycolate (TDM) extracted from such *trans*-cyclopropanase mutants stimulates inflammatory activity of murine macrophages, compared to TDM extracted from wild-type *M. tuberculosis.* Experiments like these are not feasible in humans, but the importance of MAs in human tuberculosis was recently demonstrated by implicating patient serum antibodies to mycolic acids as surrogate markers of active TB (Thanyani et al., 2008) using biosensor technology. With biosensor technology the interference of cross-reactive antibodies against cholesterol could be avoided that was encountered with the more standard ELISA technology (Schleicher et al., 2002). Here we determined that the fine structure of MAs is important for recognition by human TB patient serum antibodies.

First, we showed that methyl esters of the natural mycolate mixture showed no antigenicity, but that it was maintained by addition of a fluorescein to the MA carboxylate that substituted a new carboxylic acid functional group close to the ester bond of the conjugate. This suggested that MA assumes an antigenic configuration that is stabilized by hydrogen bonding to the (undissociated) carboxylic acid.

Two different scFv monoclonal antibody fragments generated from a chicken antibody gene library that recognized MAs, of which one cross-reacted with cholesterol and the other not, indicated that the cross-reactivity of human patient serum between MAs and cholesterol could either be due to a mixture of anti-cholesterol and anti-MA antibodies and/or due to a single antibody with cross-reactive specificity, or both. It also proves that anti-cholesterol antibodies do not necessarily cross-react with MA. Analysis of the antigenicity of a range of synthetic MA suggested that the oxygenated mycolic acids were most antigenic. Methoxy-MA represented the strongest antigen for both TB pos and TB neg patient. Important, however, was the general observation that the two functional groups on the merochain of single synthetic MAs were both critically important to determine antigenicity for human serum antibody recognition.

The results seem to favour methoxy MA of *M. tuberculosis* as the strongest functional entity or antigen to use in TB serodiagnostic devices. A proximal methyl-*trans*-cyclopropane enhances the antigenicity of these functionalities. The *R,R-*configuration of the distal methoxy group still remains to be tested in combination with a *trans*-cyclopropane proximal group to determine antigenicity in methoxy-MA. The results do not necessarily provide information as to the stereochemistry of the naturally produced MAs found in the cell walls of *M. tb*, as the antibody recognition of natural classes may be due to the complex mixtures of homologues and cyclopropane stereochemistries present. The best antigenicity seen in synthetic MA does not therefore necessarily indicate the most likely structure of the MA antigen(s) in nature. Thus, it seems unlikely that a particular MA molecule can constitute an antibody binding site filling antigen or hapten. Rather, the surface created by packed MAs is likely to be the structure that is recognized by antibodies, similar to the case of monoclonal antibody recognition of cholesterol (Kruth et al., 2001). Unlike cholesterol, which has a defined structure, MA exists as a mixture in *M. tuberculosis*. It may well be that MA folding and packing is influenced by the presence of different types of subclasses and variants thereof, such that the natural MA antigen(s) may never be recreated synthetically by the use of a single species of pure synthetic MA. It was a disappointment that the cholesterol cross-reactivity could not be defined to a particular class of MA from the antigenic mixture as implied by the observation that the TB positive and TB negative sera were not better resolved with any particular antigenic MA structure. Nevertheless, the demonstration of biological antigenic activity of individual stereochemically unique chemically synthetic MAs to levels that approximate or even exceed the antigenic activity of the natural mixture of MAs purified from *M. tuberculosis* bodes well for the possibilities towards improving the existing assays that aim at detection of anti-MA antibodies as surrogate markers for TB disease (Schleicher et al., 2002; Thanyani et al., 2008; Lemmer et al., 2009; Mathebula et al., 2009). It allows for the first time the possibility of providing exact specifications for an optimal antigen coated surface and the covalent linkage of MA to sensor surfaces for easy regeneration and engineering of the antigen to define the best window of antibody affinity and specificity.

## Figures and Tables

**Table 1:** Synthetic MA structures, names and numbers relating to results.

MA subtype	No.	Structure	Source
Alpha	**1**		Al Dulayymi et al. (2005)
	**2**		Prepared by the same methods as described in Al Dulayymi et al. (2005), but using the reverse absolute stereochemistry of the cyclopropane intermediates
Keto	**3**		Koza et al. (2009b)
	**4**		Koza et al. (2009b)
	**5**		Prepared from the corresponding protected ketone as in Koza and Baird (2007) using the methods of hydrolysis described in Koza et al. (2009b)
Methoxy	**6**		Al Dulayymi et al. (2007)
	**7**		Al Dulayymi et al. (2007)
	**8**		Al Dulayymi et al. (2007)
	**9**		Prepared using the same methods as described for the three stereoisomers above (Al Dulayymi et al., 2007)
	**10**		As for structure **5**
Hydroxy	**11**		Koza et al. (2009b)
	**12**		Koza et al. (2009b)
	**13**		As for structure **5**
	**14**		As for structure **5**
