# Erratum to “Structure–function relationships of the antigenicity of mycolic acids in tuberculosis patients” [Chem. Phys. Lipids 163 (2010) 800–808]

**DOI:** 10.1016/j.chemphyslip.2010.11.003

**Published:** 2011-02

**Authors:** Mervyn Beukes, Yolandy Lemmer, Madrey Deysel, Juma’a R. Al Dulayymi, Mark S. Baird, Gani Koza, Maximiliano M. Iglesias, Richard R. Rowles, Cornelia Theunissen, Johan Grooten, Gianna Toschi, Vanessa V. Roberts, Lynne Pilcher, Sandra Van Wyngaardt, Nsovo Mathebula, Mohammed Balogun, Anton C. Stoltz, Jan A. Verschoor

**Affiliations:** a Department of Biochemistry, University of Pretoria, South Africa; b Department of Chemistry, University of Pretoria, South Africa; c Department of Infectious Diseases, University of Pretoria, South Africa; d School of Chemistry, University of Wales, Bangor, United Kingdom; e Department of Molecular Biomedical Research, Molecular Immunology Unit, Gent University, Belgium

The publisher regrets that the author list of the above paper which appeared in *Chemistry and Physics of Lipids*, vol. 163, pp. 800–808 contained errors. The correct author list is published above.

The publisher would like to apologise for any inconvenience this may have caused to the authors of this article and readers of the journal.

