# Supplementary material for: Structure–function relationships of the antigenicity of mycolic acids in tuberculosis patients
Source: Chem Phys Lipids. 2010 Nov;163(8):800–8. doi: 10.1016/j.chemphyslip.2010.09.006 (PMC3025329; doi:10.1016/j.chemphyslip.2010.09.006)
Supplement: Supplementary file 1 [file mmc1.doc]

**(*R*)-2-((*R*)-1-Hydroxy-12-{(1*R*,2*S*)-2-[14-((1*R*,2*S*)-2-icosyl-cyclopropyl)-tetradecyl]-cyclopropyl}-dodecyl)-hexacosanoic acid.**

[α] + 2.01 °, (c = 1.2, CHCl_3_); {Found [M+Na]^+^ : 1160.59; C_78_H_152_O_3_Na requires: 1161.06}; δ_H_ (500 MHz, CDCl_3_): 3.71 (1H, m), 2.44 (1H, dt, *J* 5.05, 8.8 Hz), 1.74-1.14 (136H, m), 0.88 (6H, t, *J* 6.95 Hz), 0.65 (4H, m), 0.57 (2H, dt, *J* 4.1, 8.55 Hz), -0.32 (2H, q, *J* 5.05 Hz); δ_C_: 182.05, 72.17, 50.78, 35.54, 31.92, 30.22, 29.70, 29.66, 29.59, 29.55, 29.42, 29.36, 28.72, 27.34, 25.72, 22.68, 15.77, 14.10, 10.91; ν_max_: 3427, 3019, 2916, 2848, 1467, 1215, 759, 669 cm^-1^

**(*R*)-2-{(*R*)-17-[(1*S*,2*R*)-2-((1*S*,21*S*)-1,21-Dimethyl-20-oxo-nonatriacontyl)-cyclopropyl]-1-hydroxy-heptadecyl}-hexacosanoic acid**

[α] + 4.74 (*c* 0.78, CHCl_3_), m.p.: 66–68 ^o^C, {Found (M + Na)^+^: 1302.2935, C_87_H_170_NaO_4_ requires: 1302.2991}; δ_H_: 3.74–3.70 (1H, m), 2.51 (1H, sext, *J* 6.9 Hz), 2.46 (1H, dt, *J* 8.8, 5.4 Hz), 2.43 (1H, dt, *J* 14.8, 7.3 Hz), 2.40 (1H, dt, *J* 14.8, 7.3 Hz), 1.78–1.14 (148H, m, v.br.), 1.05 (3H, d, *J* 6.9 Hz), 0.90 (3H, d, *J* 6.9 Hz), 0.89 (6H, t, *J* 6.9 Hz), 0.71–0.64 (1H, m), 0.48–0.42 (1H, m), 0.22–0.08 (3H, m); δ_C_: 215.4, 180.0, 72.1(-), 50.9(-), 46.3(-), 41.1(+), 38.1(-), 37.4(+), 35.5(+), 34.5(+), 33.0(+), 31.9(+), 30.1(+), 29.7(+, v.br.), 29.66(+), 29.61(+), 29.52(+), 29.50(+), 29.47(+), 29.44(+), 29.37(+), 29.34(+), 27.3(+), 27.2(+), 26.1(-), 25.7(+), 23.7(+), 22.7(+), 19.7(-), 18.6(-), 16.3(-), 14.1(-), 10.5(+); ν_max_: 3348, 2919, 2850, 1697, 1471, 1376, 1185, 1023 cm^-1^.

**(*R*)-2-[(*R*)-1-Hydroxy-18-[(1*S*,2*R*)-2((17*S*,18*S*)-17-methoxy-18-methylhexatriacontyl)cyclopropyl]octadecyl]-hexacosnoic acid**

[α] +1.17 (CHCl_3_, 0.496 µmol); m.p. 65-67°C; [Found M+Na^+^: 1276.12; C_85_H_168_NaO_4_ requires: 1276.28]; δ_H_ (500 MHz, CDCl_3_): 3.77-3.69 (1H, m), 3.35 (3H, s), 2.99-2.96 (1H, m), 2.46 (1H, dt, *J* 5.26, 9.31), 1.78-1.70 (1H, m), 1.68-1.60 (2H, m), 1.56-1.03 (146H, br m including br s at 1.26), 0.89 (6H, t, *J* 6.86 Hz), 0.85 (3H, d, *J* 6.9 Hz), 0.68-0.62 (2H, m), 0.56 (1H, dt, *J* 4, 8.15 Hz), -0.32 (1H, dt, *J* 5 Hz); δ_C_ (125 MHz, CDCl_3_): 178.9, 85.6, 72.1, 57.7, 50.7, 35.6, 35.3, 32.4, 31.9, 30.5, 30.3, 30.2, 29.9, 29.7, 29.7, 29.6, 29.6, 29.5, 29.4, 29.4, 28.7, 27.6, 27.3, 26.1, 22.7, 18.0, 15.8, 14.9, 14.1, 10.9; v_max_: 3521, 2923, 2854, 1713, 1459 cm^-1^.

**(*R*)-2-((*R*)-1-Hydroxy-19-((1*S*,2*R*)-2-((2*S*,18*S*,19*S*)-18-methoxy-19-methylheptatriacontan-2-yl)cyclopropyl)nonadecyl)hexacosanoic acid**

[α] -0.97 (CHCl_3_, 0.468 µmol); m.p. 70-72°C; [Found M+Na^+^: 1318.24; C_88_H_174_NaO_4_ requires: 1318.33]; δ_H_ (500 MHz, CDCl_3_): 3.74-3.70 (1H, m), 3.36 (3H, s), 2.99-2.96 (1H, m), 2.49-2.45 (1H, m), 1.79-1.72 (1H, m), 1.69-1.61 (2H, br m), 1.55-1.10 (149H, br m including br s at 1.27), 0.91-0.85 (12H, br m including t, *J* 6.95 Hz), 0.48-0.43 (1H, m), 0.22-0.18 (1H, m), 0.17-0.14 (1H, m), 0.13-0.09 (1H, m); δ_C_ (125 MHz, CDCl_3_): 178.7, 85.6, 72.1, 65.9, 57.7, 50.6, 38.2, 37.4, 35.6, 35.3, 34.5, 32.4, 31.9, 30.5, 30.1, 30.0, 29.9, 29.72, 29.69, 29.64, 29.59, 29.5, 29.43, 29.39, 27.6, 27.3, 27.2, 26.2, 25.8, 22.7, 19.7, 18.6, 15.3, 14.9, 14.1, 10.5; v_max_: 3520, 2924, 2853, 1735, 1465 cm^-1^.

**(*R*)-2-{(*R*)-1-Hydroxy-17-[(1*S*,2*R*)-2-((1*S*,20*S*,21*S*)-20-hydroxy-1,21-dimethyl-nonatriacontyl)-cyclopropyl]-heptadecyl}-hexacosanoic acid**

[α] -1.04 (*c* 0.54, CHCl_3_), m.p.: 51–53 ^o^C, {Found (M + Na)^+^: 1304.3084, C_87_H_172_NaO_4_ requires: 1304.3148}; δ_H_: 3.73–3.69 (1H, m), 3.53–3.50 (1H, m), 2.46 (1H, dt, *J* 8.8, 5.4 Hz), 1.75–1.59 (1H, m), 1.55–1.12 (151H, m, v.br.), 0.91–0.86 (12H, m), 0.69–0.63 (1H, m), 0.48–0.42 (1H, m), 0.22–0.08 (3H, m); δ_C_: 178.8, 75.4(-), 72.1(-), 50.7(-), 38.2(-), 38.1(-), 37.4(+), 35.6(+), 34.5(+), 34.4(+), 33.3(+), 31.9(+), 30.1(+), 30.0(+), 29.73(+), 29.71(+, v.br.), 29.66(+), 29.60(+), 29.54(+), 29.43(+), 29.36(+), 27.4(+), 27.3(+), 27.2(+), 26.3(+), 26.1(-), 25.7(+), 22.7(+), 19.7(-), 18.6(-), 14.1(-), 13.6(-), 10.5(+); ν_max_: 3331, 2920, 2851, 1686, 1460, 1377, 1201 cm^-1^.

**(*R*)-2-{(*R*)-1-Hydroxy-17-[(1*S*,2*R*)-2-((1*S*,20*R*,21*R*)-20-hydroxy-1,21-dimethyl-nonatriacontyl)-cyclopropyl]-heptadecyl}-hexacosanoic acid**

[α] +11.4 (*c* 0.60, CHCl_3_); {Found (M + Na)^+^: 1304.19, C_87_H_172_NaO_4_ requires: 1304.31}; δ_H_: 3.73–3.69 (1H, m), 3.53–3.50 (1H, m), 2.46 (1H, dt, *J* 8.8, 5.4 Hz), 1.75–1.59 (1H, m), 1.55–1.12 (151H, m, v.br.), 0.91–0.86 (12H, m), 0.69–0.63 (1H, m), 0.48–0.42 (1H, m), 0.22–0.08 (3H, m); δ_C_: 178.8, 75.4(-), 72.1(-), 50.7(-), 38.2(-), 38.1(-), 37.4(+), 35.6(+), 34.5(+), 34.4(+), 33.3(+), 31.9(+), 30.1(+), 30.0(+), 29.73(+), 29.71(+, v.br.), 29.66(+), 29.60(+), 29.54(+), 29.43(+), 29.36(+), 27.4(+), 27.3(+), 27.2(+), 26.3(+), 26.1(-), 25.7(+), 22.7(+), 19.7(-), 18.6(-), 14.1(-), 13.6(-), 10.5(+); ν_max_: 3331, 2920, 2851, 1686, 1460, 1377, 1201 cm^-1^.
